# Supplementary material for: Spatiotemporal dynamics and heterogeneity of renal lymphatics in mammalian development and cystic kidney disease
Source: eLife. 2019 Dec 6;8:e48183. doi: 10.7554/eLife.48183 (PMC6948954; doi:10.7554/eLife.48183)
Supplement: Source code 1. [file elife-48183-code1.docx]

// Script for segmentation of PROX1+/LYVE-1+ structures as used in Jafree et al. eLife 2019, written in JAVA format and used in ImageJ / FIJI

// Take nuclei from Ch1, vessels from Ch2, segment each and use Morphological reconstruction

// Generates an output with just LYVE-1+ vessels with PROX1+ overlapping nuclei

// Created by Dale Moulding (UCL GOSICH Light Microscopy facility) & modified by Daniyal Jafree. August 2019

// Segment PROX1+ nuclei

run("Duplicate...", "title=orig duplicate");

run("Duplicate...", "title=cells duplicate channels=1");

run("Subtract Background...", "rolling=50 stack");

setAutoThreshold("RenyiEntropy dark stack");

run("Convert to Mask", "method=RenyiEntropy background=Dark");

run("3D Simple Segmentation", "low_threshold=128 min_size=50 max_size=10000"); //set nuclei size, requires tweaking

setThreshold(1, 65535);

setOption("BlackBackground", false);

run("Convert to Mask", "method=Default background=Dark");

run("Invert LUT");

rename("cellsmask");

selectWindow("Bin");

close();

selectWindow("cells");

close();

// Segment LYVE-1+ structures

selectWindow("orig");

run("Duplicate...", "title=vessels duplicate channels=2");

run("Subtract Background...", "rolling=50 stack");

setAutoThreshold("Moments dark stack");

run("Convert to Mask", "method=Moments background=Dark");

run("3D Simple Segmentation", "low_threshold=128 min_size=100 max_size=1000000");// set vessel sizes

setThreshold(1, 65535);

setOption("BlackBackground", false);

run("Convert to Mask", "method=Default background=Dark");

run("Invert LUT");

rename("vesselsmask");

selectWindow("Bin");

close();

selectWindow("vessels");

close();

// Exclude non overlapping regions using IJPB plugin Morpholibj Morphological reconstruction

setBatchMode(true);

selectWindow("cellsmask");

n = nSlices();

for (i=1; i<=n; i++) {

showProgress(i, n);

selectWindow("cellsmask");

setSlice(i);

selectWindow("vesselsmask");

setSlice(i);

run("Morphological Reconstruction", "marker=cellsmask mask=vesselsmask type=[By Dilation] connectivity=4");

if (i==1)

output = getImageID();

else {

run("Select All");

run("Copy");

close();

selectImage(output);

run("Add Slice");

run("Paste");

}

}

run("Select None");

setBatchMode(false);

// Create a new image of the segmented PROX1+/LYVE-1+ vessels on top of the original

selectWindow("orig");

run("Split Channels");

run("Merge Channels...", "c1=C1-orig c2=C2-orig c4=cellsmask-rec create");

selectWindow("vesselsmask");

close();

selectWindow("cellsmask");

close();
